# Parental education and income are linked to offspring cortical brain structure and psychopathology at 9–11 years

**DOI:** 10.1002/jcv2.12220

**Published:** 2024-02-06

**Authors:** Linn B. Norbom, Jaroslav Rokicki, Espen M. Eilertsen, Thea Wiker, Jamie Hanson, Andreas Dahl, Dag Alnæs, Sara Fernández‐Cabello, Dani Beck, Ingrid Agartz, Ole A. Andreassen, Lars T. Westlye, Christian K. Tamnes

**Affiliations:** ^1^ PROMENTA Research Center Department of Psychology University of Oslo Oslo Norway; ^2^ NORMENT Institute of Clinical Medicine University of Oslo Oslo Norway; ^3^ Centre of Research and Education in Forensic Psychiatry Oslo University Hospital Oslo Norway; ^4^ Department of Psychiatric Research Diakonhjemmet Hospital Oslo Norway; ^5^ Learning Research and Development Center University of Pittsburgh Pennsylvania Pittsburgh USA; ^6^ Department of Psychology University of Pittsburgh Pennsylvania Pittsburgh USA; ^7^ Department of Psychology University of Oslo Oslo Norway; ^8^ Department of Psychology Pedagogy and Law Kristiania University College Oslo Norway; ^9^ K.G Jebsen Center for Neurodevelopmental Disorders University of Oslo Oslo Norway; ^10^ Centre for Psychiatry Research Department of Clinical Neuroscience Karolinska Institutet & Stockholm Health Care Services Stockholm Sweden; ^11^ NORMENT Division of Mental Health and Addiction Oslo University Hospital & Institute of Clinical Medicine University of Oslo Oslo Norway

**Keywords:** cortical morphometry, development, grey‐/white‐matter contrast (GWC), linked independent component analysis (LICA), MRI, multimodal fusion, socioeconomic status (SES)

## Abstract

**Background:**

A child's socioeconomic environment can shape central aspects of their life, including vulnerability to mental disorders. Negative environmental influences in youth may interfere with the extensive and dynamic brain development occurring at this time. Indeed, there are numerous yet diverging reports of associations between parental socioeconomic status (SES) and child cortical brain morphometry. Most of these studies have used single metric‐ or unimodal analyses of standard cortical morphometry that downplay the probable scenario where numerous biological pathways *in sum* account for SES‐related cortical differences in youth.

**Methods:**

To comprehensively capture such variability, using data from 9758 children aged 8.9–11.1 years from the ABCD Study^®^, we employed linked independent component analysis (LICA) and fused vertex‐wise cortical thickness, surface area, curvature and grey‐/white‐matter contrast (GWC). LICA revealed 70 uni‐ and multimodal components. We then assessed the linear relationships between parental education, parental income and each of the cortical components, controlling for age, sex, genetic ancestry, and family relatedness. We also assessed whether cortical structure moderated the negative relationships between parental SES and child general psychopathology.

**Results:**

Parental education and income were both associated with larger surface area and higher GWC globally, in addition to local increases in surface area and to a lesser extent bidirectional GWC and cortical thickness patterns. The negative relation between parental income and child psychopathology were attenuated in children with a multimodal pattern of larger frontal‐ and smaller occipital surface area, and lower medial occipital thickness and GWC.

**Conclusion:**

Structural brain MRI is sensitive to SES diversity in childhood, with GWC emerging as a particularly relevant marker together with surface area. In low‐income families, having a more developed cortex across MRI metrics, appears beneficial for mental health.

## Graphical abstract

An exploration of the relationship between parental socioeconomic status (SES) and child cortical brain structure. Using data from 9758 children aged 8.9–11.1 years, results showed that parental education and income were associated with larger surface area and higher gray/white‐matter contrast (GWC) globally, as well as local increases in surface area and bidirectional GWC and cortical thickness patterns. Furthermore, having a more developed cortex across MRI metrics was beneficial for mental health in low‐income families.

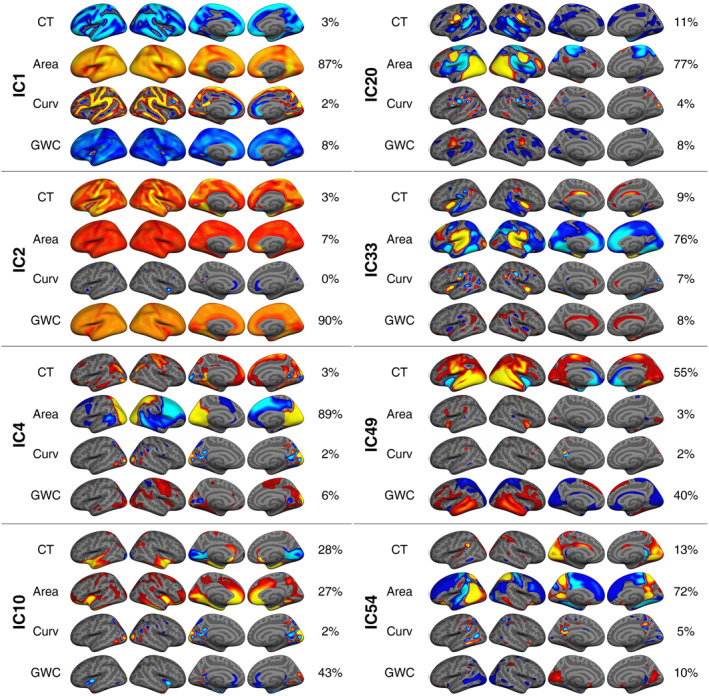

**Key Points**

- What is known: Children's socioeconomic environment can shape central aspects of their life, including the risk of mental disorders, and negative influences may interfere with brain development during this time.
- What is new: The study leverages the ABCD Study and linked independent component analysis to reveal associations between parental socioeconomic status (SES) and child cortical morphology and microstructure. It identifies grey‐/white‐matter contrast (GWC) and cortical surface area as the most relevant markers. The negative relation between parental income and child psychopathology were attenuated in children with a multimodal developmental pattern.
- What is relevant: The study underscores the importance of considering SES diversity not just in developmental research, but also in policy formulation. Efforts should be aimed at narrowing SES gaps and providing targeted interventions in low‐SES communities, thereby fostering positive development in childhood and mental health

## INTRODUCTION

The socioeconomic environment of a child can shape many central aspects of their life, including life expectancy, present and prospective cognitive abilities, school performance, and susceptibility for mental health struggles (Thomas & Coecke, 2023). Childhood is also marked by extensive brain development. These neuronal optimization processes, caused by genetic and environmental factors in complex interplay, are indirectly detectable by magnetic resonance imaging (MRI) (Jernigan et al., 2016; Lebel & Deoni, 2018; Norbom et al., 2021). Although brain plasticity fosters adaptation and learning, by the same token, environmental variables including socioeconomic status (SES) can affect brain development and influence the risk of mental disorders (Dearing et al., 2006; Farah, 2017; Letourneau et al., 2013).

As theorized by sociologist Pierre Bourdieu, SES is a complex dimensional construct used to assess social (connections), cultural (skills, knowledge and education), symbolic (prestige), and financial capital (Bourdieu, 2011).Within neuroscience, SES is often assessed by material gains like income, and non‐material gains such as education and occupation (Long & Renbarger, 2023). This tradition has been critiqued and expanded to include subjective and individual experiences of social class (W. M. Liu et al., 2004), and cultural knowledge and abilities of marginalized minority groups that often go unrecognized (Yosso, 2005).

Low parental SES is associated with a broad array of negative outcomes in children. This includes lower cognitive abilities, a discrepancy that widens across childhood (Duyme et al., 1999; von Stumm & Plomin, 2015; Zhang et al., 2020), and poorer academic achievements (Sirin, 2005). Youths from lower SES families are also 2–3 times more likely to suffer from mental health problems than their higher SES peers (Letourneau et al., 2013; Reiss, 2013). Although the association between parental SES and youth psychopathology is established, how the brain affects these relations, remain poorly understood. SES is also heritable (Hill et al., 2019; Tambs et al., 2012; Ørstavik et al., 2014) complicating the causal relations with brain and mental health.

Childhood is a central period for brain maturation, involving multiple biological processes that show spatial and temporal heterogeneity across tissue types, metrics, and individuals (Jernigan et al., 2016; Lebel & Deoni, 2018; Norbom et al., 2021). The cerebral cortex shows a particularly lengthy developmental trajectory, with protracted decreases in apparent thickness and early increases in surface area and curvature (Norbom et al., 2021; Sydnor et al., 2021). Beyond morphometry, a prominent feature of youth development is an increase in cortical brightness. Variations in cortical brightness can be assessed through T1‐weighted (T1w) intensity metrics such as the gray/white‐matter contrast (GWC) (Salat et al., 2009). As cholesterol in myelin is a major determinant of the T1w‐signal (Koenig, 1991; Koenig et al., 1990), GWC has been suggested as a viable proxy for intracortical myelination (Jørgensen et al., 2016), a crucial feature of postnatal brain development, allowing for efficient signal transmission and structural support (Bartzokis, 2012; Baumann & Pham‐Dinh, 2001; S. Liu et al., 2019; Waxman & Bennett, 1972).

There are several reports of associations between parental SES and child cortical structure (Khundrakpam et al., 2020; Noble et al., 2015; Piccolo et al., 2016; Rakesh et al., 2022; Tomasi & Volkow, 2021). Findings are discrepant both in terms of the sensitivity of different imaging modalities, and the direction. Still, a recent comprehensive review of 71 studies pointed to quite consistent positive relations between parental SES and global as well as frontal surface area in childhood (Rakesh & Whittle, 2021). The review found no studies employing intensity metrics like GWC, or cortical curvature, but a single study on the related metric gyrification. The general divergence in findings could partly be explained by varying SES measures and distinct SES subfactor ‐ cortical metric relationships (Farah, 2017; Rakesh & Whittle, 2021). Also, due to convention and availability, many recent studies use imaging ROI's from Desikan‐Killiany or the Destrieux Atlas (Desikan et al., 2006; Destrieux et al., 2010). These atlases are not based on cyto‐ or myeloarchitecture, which are the main proposed neurobiological drivers of cortical thickness, area and GWC, but instead on gyral folding. Rather than adhering to a priori divisions, one could perform a data driven reduction of the vertex‐wise data. A related challenge is that most studies have assessed a single‐, or a few selected morphometric measures separately. This downplays the probable scenario of complex constructs like SES affecting numerous biological pathways with unique genetic and environmental determinants (Hogstrom et al., 2013; Rakic, 1988; Strike et al., 2019) that in sum underlie the SES—cortical structure relationship.

Multivariate imaging approaches (Groves et al., 2011; Miller et al., 2016) can co‐model several sources of variability, which could increase effect sizes and improve neurobiological interpretation. For instance, while an independent component analysis (ICA) decomposes a signal into its constituent parts, linked ICA (LICA) can simultaneously “link”, or model common features across modalities irrespective of the units' signal‐ and contrast‐to‐noise ratios and spatial smoothness (Groves et al., 2011). LICA studies have reported unique structural patterns sensitive to brain development and psychopathology (Groves et al., 2012; Norbom et al., 2020; Wolfers et al., 2017). Moreover, a recent study using canonical correlation analysis (CCA) reported common modes capturing SES factors and patterns of cortical morphometry, including sulcal depth (Alnæs et al., 2020). Thus, while it is reasonable to use multimodal reduction approaches that go beyond standard morphometry by including cortical curvature and GWC for a comprehensive assessment of the parental SES and child cortical structure relationship, this has not previously been done.

Using data obtained from 9758 children aged 8.9–11.1 years from the Adolescent Brain Cognitive Development (ABCD) Study^®^, we used LICA to perform multimodal fusion of vertex‐wise cortical thickness, surface area, curvature and GWC. We then assessed the linear relation between parental education and parental income and each LICA component, and whether components could moderate the negative relationship between parental SES and general psychopathology. We hypothesized that parental education and income would show positive associations with global and frontal surface area (Noble et al., 2015; Rakesh & Whittle, 2021; Thomas & Coecke, 2023), and with widespread GWC for parental education (Norbom et al., 2022). We expected no parental SES ‐ cortical thickness relations (Norbom et al., 2022; Rakesh & Whittle, 2021), and had no specific hypotheses pertaining to curvature or multimodal coupling.

## MATERIALS AND METHODS

### Participants

Data was acquired from the ABCD Study^®^ using the curated annual release 4.0 (https://data‐archive.nimh.nih.gov/abcd), with further detail described in the Supplemental Information (SI). The ABCD study^®^ consists of data from almost 12,000 children, aged approximately 9–10 years at study inclusion, as well as their parents. The data is obtained across 21 sites in the United States of America and includes detailed demographic, genetic, behavioral and neuroimaging data that will be collected for a decade (Feldstein Ewing et al., 2018).

The current study was based on the baseline assessment of the ABCD study^®^. From 11,876 participants, 8 children had partly, or completely missing Child Behavior Checklist (CBCL) raw scores and were therefore excluded. 14 children with missing educational data for both parents and 631 children with completely missing parental income information were also excluded. Another 732 children were excluded due to missing genetic ancestry data. On the imaging side we had FreeSurfer processed data for 11,591 individuals. Of these 535 individuals did not pass MRI quality control or had missing data (see below) and were therefore excluded. 565 subjects were excluded during the intersection of individuals with complete demographics and complete neuroimaging resulting in a final sample size of 9758 participants (5124 females) aged 8.9–11.1 years (mean = 9.9, SD = 0.6), including 18 triplets and 1662 mono‐ and dizygotic twins.

### Measurement of socioeconomic status

Socioeconomic information was reported by a parent or guardian on behalf of themselves and a partner if relevant, by completing the “ABCD parental demographics survey”.

Parental education was assessed with the question “What is the highest grade/level/degree you have completed or received” ranging from 0 = Never attended/Kindergarten only, to 21 = Doctoral degree. We recoded this variable to years of total education as described in SI and defined parental education as the highest educational score of either the reporting parent or their partner.

Parental income was assessed with the question “How much did you earn, before taxes and other deductions, during the past 12 months?”, ranging from 1 = Less than $5.000, to 10 = $200.000 or greater. Total family income was assessed with the question “What is your total combined family income for the past 12 months?” using identical scoring. We recoded these variables using the median of each bracket as described in the SI and defined parental income as the highest number available from parent, partner, or combined income. The distributions of raw SES scores are shown in SI Figure 1.

### Assessment of general psychopathology

Child psychopathology was assessed using the CBCL (Achenbach & Ruffle, 2000), which is a widely used caregiver report for identifying behavioral and emotional problems in children. The CBCL contains 119 items pertaining to particular behaviors, and the reporter must assess, on a 3‐point scale, to which extent these are characteristic of the child during the past six months. We calculated an overarching “p‐factor” in accordance with “the general factor of psychopathology model seven” from Clark et al. (2021) where items were first grouped into three lower order factors, namely internalizing, externalizing and attention problems before being summed to a higher order p‐factor. P‐factor distribution within our final sample is presented in SI Figure 2.

### MRI acquisition, quality control, preprocessing and scanner harmonization

MRI data was attained on 29 different 3T scanners from Siemens Prisma, General Electric (GE) 750 and Philips. The T1w image was an inversion prepared RF‐spoiled gradient echo scan, using prospective motion correction when available, and with 1 mm isotropic voxel resolution. Detailed descriptions of acquisition parameters, and care and safety procedures implemented for scanning of children are presented elsewhere (Casey et al., 2018).

We relied on the T1w quality control from the ABCD Data Analysis and Informatics Core which uses a standardized pipeline of automated and manual procedures (Hagler et al., 2019). It yields a binary code for images recommended for inclusion, and 367 youths did not pass ABCD QC and were therefore excluded. Post FreeSurfer processing (see below) we additionally excluded 104 subjects due to missing surface‐based data, and 64 subjects due to total amount of surface holes being => 200 before correction (Elyounssi et al., 2023; Rosen et al., 2018).

Quality approved T1w images were processed using the open‐source neuroimaging toolkit FreeSurfer 7.1 (http://surfer.nmr.mgh.harvard.edu). FreeSurfer performs volumetric segmentations and cortical surface reconstructions, including the “white” and “pial” surface, which is the gray/white matter boundary and the gray/cerebrospinal fluid (CSF) boundary, respectively (Dale et al., 1999; Fischl et al., 1999). The computation of each imaging metric including GWC is described in detail within SI. Within the current study lower GWC reflects more similar gray and white matter, a blurring that is documented across youth development (Norbom et al., 2019). Thickness, area, curvature and GWC surface maps were registered to fsaverage and smoothed using a Gaussian kernel of 15 mm full width at half maximum (FWHM).

To adjust for systematic and unwanted scanner related variance, smoothed surfaces were subsequently imported to R and the package neuroCombat (Fortin et al., 2018) was employed at vertex level to harmonize data across scanners. We included five covariates to our Combat model, namely age, sex, p‐factor, parental income, and parental education, to preserve such variance during the harmonization procedure. Box plots of mean or total MRI measures pre‐ and post‐neuroCombat adjustments are presented in SI Figuress 3–6.

### Multimodal fusion

Combat corrected vertex‐wise surfaces of cortical thickness, surface area, curvature and GWC were fused by FMRIB's Linked Independent Component Analysis (FLICA) (Groves et al., 2011). FLICA decomposes data into spatially independent components of variation and is robust to inputs of differing units, smoothness, and signal‐ and contrast to noise ratios. It can discover both multimodal features, and detect single‐modality structured components if present (Groves et al., 2011, 2012). The FLICA mixing matrix vectors are statistically independent but not required to be orthogonal and can therefore account for shared variance from variables external to the FLICA. FLICA was employed with 1000 iterations, and a log‐transform of surface area only. A model order of 70 was chosen based on having the highest cophenetic correlation coefficient after testing model orders ranging from 50 to 80. This range was chosen to balance coherent statistical analyses and interpretable findings with the ability to discern distinct patterns. The cophenetic correlation coefficient is an indication of how well the similarities of the clustering result matches subject resemblances within the original dataset.

### Statistical analyses

**FIGURE 1:**
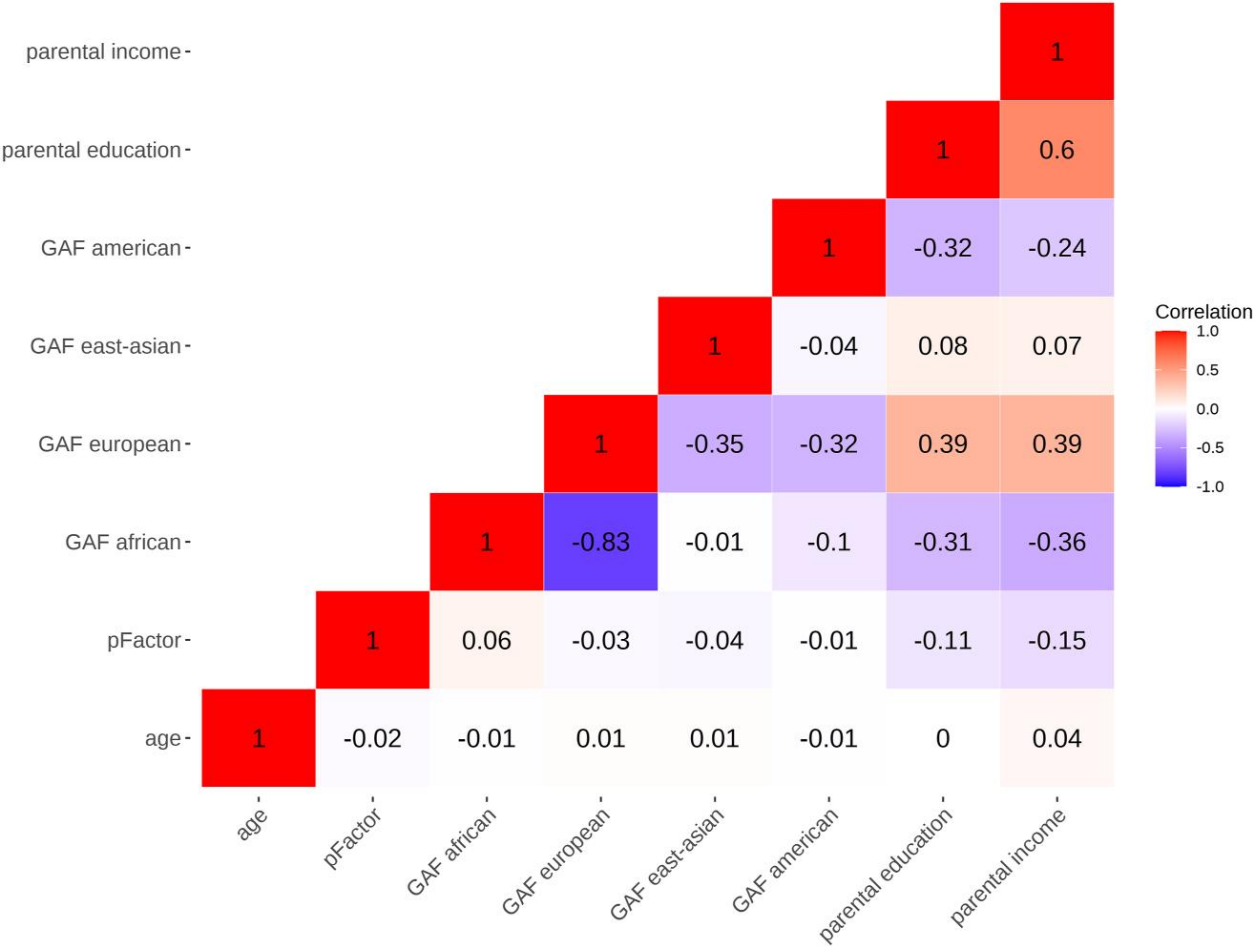
Pearson's correlations of key demographic and behavioral variables. The figure shows a correlation matrix of all the demographic and behavioral variables from the final sample (*n* = 9758), which were added as fixed effects and dependent variables for our main analyses.

All demographic and behavioral data (see Figure 1 for a correlation matrix) were z‐standardized, and associations between parental SES and LICA component subject loadings were tested using linear mixed effects (LME) models in R. We used the “lme4” (Bates et al., 2015), and the “lme.dscore” package from EMAtools, the latter for Cohens D calculations.

First, we tested the linear association between parental education and the 70 LICA components in separate models. Parental education and subject loading were added as independent and dependent variables, respectively. Age, sex, and four genetic ancestry factors (GAFs) were included as fixed effects, the latter based on population inference from genetic variants (Huang et al., 2022), and were included to minimize confounds from population stratification. To maximize generalizability and statistical power, we included family ID and monozygotic twin status as random effects, to model shared environmental and genetic influences. We then ran identical analyses testing the linear association between parental income and each LICA component. Tests of the quadratic relationship between both parental SES metrics and LICA components are presented in the SI.

Second, we tested the linear association between parental SES and child p‐factor scores. We included parental education or income as independent variables in separate models and p‐factor scores as the dependent variable. Fixed and random effects were identical to our previous models.

Third, we tested whether relevant LICA components (defined as showing a significant association with parental SES), could moderate the relationship between parental SES and child p‐factor scores. The interaction term between parental education and subject loading was included as an independent variable, while child p‐factor was added as a dependent variable. Parental education, subject loading, age, sex, and GAFs were added as fixed effects, while family ID and monozygotic twin status were added as random effects. We then ran identical analyses where parental education was replaced with parental income.

Finally, to compare our multimodal results to more conventional unimodal approaches, we tested the linear association between parental SES and standardized mean cortical thickness, total surface area, mean curvature, and mean GWC in separate models.

For all statistical analyses with multiple comparisons, *p*‐values were adjusted by false discovery rate (FDR) using Benjamini‐Hochberg's procedure and a significance threshold of 0.05. The statistical code used in the present paper can be found online (https://osf.io/etsjx/).

## RESULTS

### Multimodal decomposition

**FIGURE 2:**
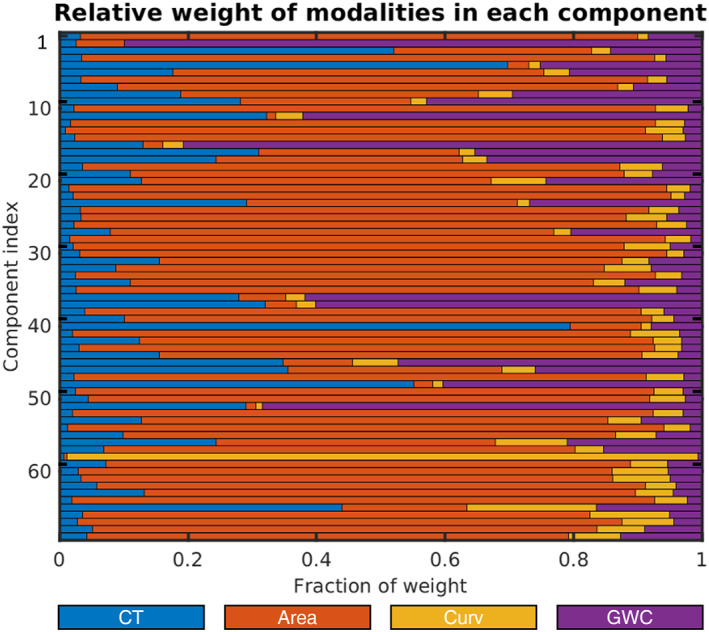
Linked independent component analysis (LICA) decomposition. The figure shows the color‐coded relative weight of cortical thickness (CT), surface area (Area), curvature (Curv) and gray/white‐matter contrast (GWC), within each of the 70 components.

FLICA decomposition resulted in 70 independent components (ICs) of youth cortical brain structure. Modal weighting was dominated by surface area followed by GWC, cortical thickness, and with relatively little contribution from curvature, as visualized in Figure 2. Of note, IC59, the only component showing a strong curvature weighting was highly driven by a single subject and was therefore disregarded. ICs were ordered based on total explained variance with IC1 and IC2 explaining 18.7% and 16% respectively. Both ICs were mainly unimodal, the first highly dominated by global surface area and the second by global GWC. The remaining components explained between 3.23% and 0.47% of total variance in the cortical decomposition, and all components should be interpreted as showing patterns beyond, or “in addition” to the other components.

### Linear associations between parental education and youth cortical structure

**FIGURE 3:**
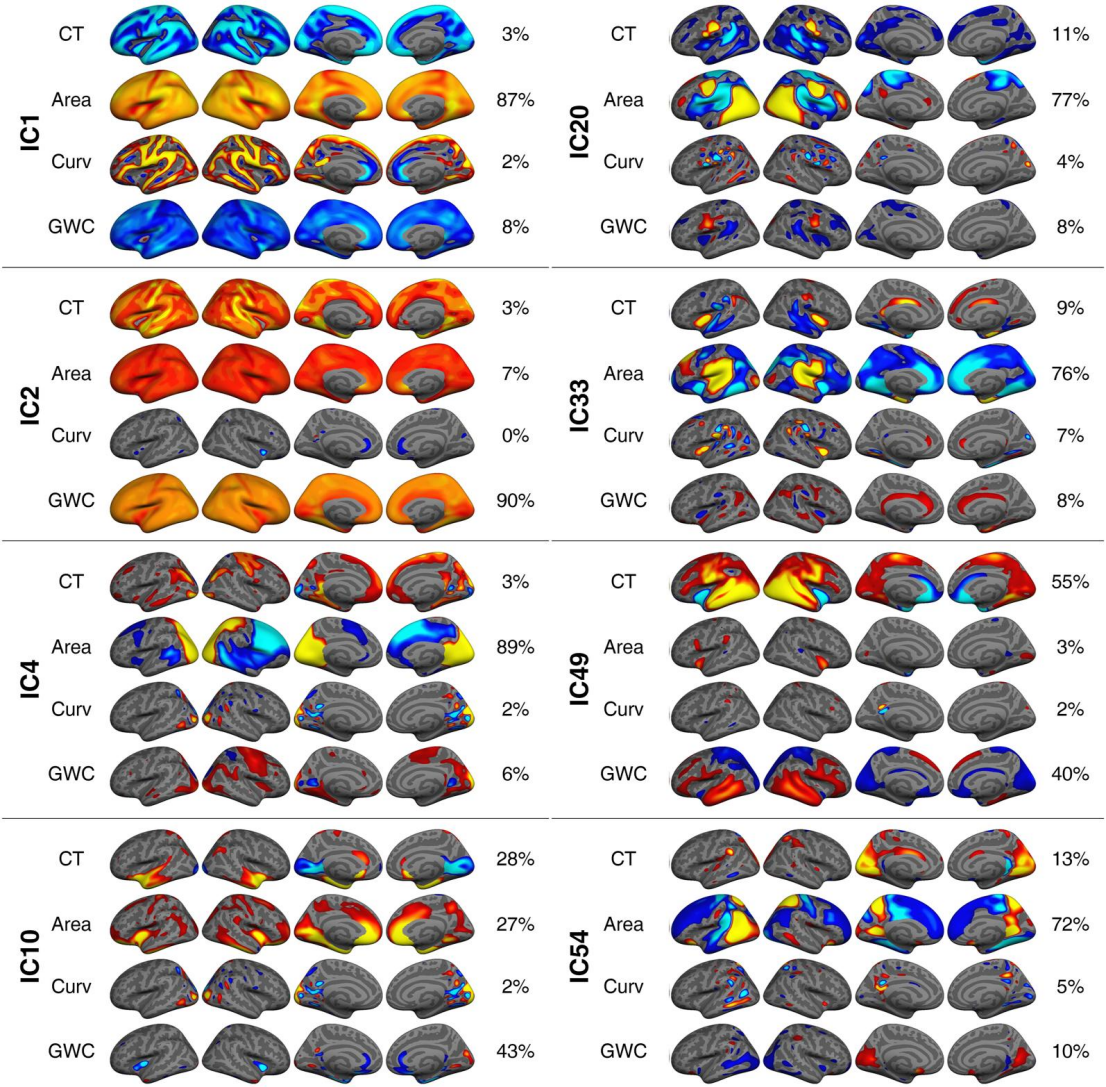
Spatial visualization of independent components (ICs) showing a significant association with parental education and/or income. All components were thresholded with a minimum and maximum of 7 and 20 standard deviations, respectively, except for surface area within IC1 and IC2, and GWC within IC2, which were thresholded with a higher value to reveal nuances in the global pattern.

LME models revealed significant associations between parental education and six ICs of youth cortical structure, namely IC1, IC2, IC10, IC20, IC49 and IC54 (see Table 1 and Figure 3).

**TABLE 1:** Associations between parental socioeconomic status (SES) and cortical component loadings.

	Parental education				Parental income			
IC	Cohens D	T Statistic	*p*‐value (uncorrected)	*p*‐value (corrected)	Cohens D	T Statistic	*p*‐value (uncorrected)	*p*‐value (corrected)
1	0.1	4.52	<0.000*	<0.000*	0.09	4.25	<0.000*	0.001*
2	0.13	5.8	<0.000*	<0.000*	0.15	6.72	<0.000*	<0.000*
3	0.06	2.7	0.007*	0.411	0.05	2.4	0.016*	0.952
4	−0.07	−3	0.003*	0.167	−0.09	−3.81	<0.000*	0.009*
5	0	−0.07	0.947	0.997	−0.03	−1.32	0.186	0.974
6	−0.01	−0.49	0.628	0.997	0	−0.13	0.896	0.974
7	−0.07	−3.24	0.001*	0.074	−0.03	−1.42	0.156	0.974
8	0	−0.02	0.982	0.997	0.02	0.78	0.434	0.974
9	0	0	0.997	0.997	0.03	1.33	0.185	0.974
10	0.14	6.17	<0.000*	<0.000*	0.11	5.08	<0.000*	<0.000*
11	0.01	0.38	0.704	0.997	−0.01	−0.26	0.794	0.974
12	−0.02	−0.9	0.37	0.997	0.01	0.33	0.738	0.974
13	0.03	1.38	0.169	0.997	0.02	0.86	0.388	0.974
14	−0.03	−1.2	0.23	0.997	−0.01	−0.52	0.606	0.974
15	−0.04	−1.6	0.11	0.997	−0.04	−1.56	0.118	0.974
16	0.02	1.06	0.291	0.997	0.01	0.62	0.537	0.974
17	−0.03	−1.49	0.136	0.997	−0.05	−2.35	0.019*	0.974
18	−0.01	−0.64	0.522	0.997	−0.01	−0.63	0.528	0.974
19	−0.01	−0.47	0.635	0.997	−0.05	−2.12	0.034*	0.974
20	0.09	4.13	<0.000*	0.002*	0.08	3.8	<0.000*	0.01*
21	0.04	2.02	0.044*	0.997	0.07	3.23	0.001*	0.077
22	0.05	2.42	0.016*	0.899	0.02	0.93	0.352	0.974
23	−0.01	−0.34	0.732	0.997	0.01	0.35	0.729	0.974
24	0.02	0.95	0.34	0.997	0.02	1.01	0.311	0.974
25	−0.06	−2.64	0.008*	0.486	−0.05	−2.3	0.021*	0.974
26	−0.04	−1.62	0.106	0.997	−0.03	−1.26	0.207	0.974
27	0	0.07	0.946	0.997	−0.03	−1.49	0.136	0.974
28	−0.02	−0.7	0.483	0.997	−0.03	−1.36	0.175	0.974
29	−0.05	−2.22	0.027*	0.997	0	0.17	0.864	0.974
30	−0.02	−0.68	0.495	0.997	−0.02	−0.97	0.331	0.974
31	0.04	1.61	0.108	0.997	0	0.15	0.882	0.974
32	0.01	0.27	0.791	0.997	0.03	1.2	0.23	0.974
33	0.04	1.77	0.076	0.997	0	−0.05	0.964	0.974
34	0.01	0.41	0.681	0.997	0.01	0.43	0.671	0.974
35	−0.01	−0.29	0.775	0.997	−0.02	−0.72	0.472	0.974
36	−0.01	−0.25	0.8	0.997	0.01	0.5	0.618	0.974
37	−0.03	−1.42	0.156	0.997	−0.01	−0.56	0.579	0.974
38	0	−0.17	0.866	0.997	−0.02	−1.05	0.295	0.974
39	−0.04	−1.97	0.049*	0.997	−0.02	−0.69	0.492	0.974
40	−0.01	−0.36	0.718	0.997	0	−0.22	0.828	0.974
41	−0.06	−2.8	0.005*	0.313	−0.03	−1.31	0.191	0.974
42	0	−0.16	0.872	0.997	−0.01	−0.49	0.624	0.974
43	−0.03	−1.31	0.191	0.997	−0.05	−2.24	0.025	0.974
44	−0.03	−1.49	0.136	0.997	0	0.03	0.974	0.974
45	−0.04	−1.82	0.069	0.997	−0.07	−3.28	0.001*	0.065
46	−0.03	−1.31	0.192	0.997	0.04	1.88	0.061	0.974
47	0	−0.06	0.952	0.997	0.05	2.07	0.039*	0.974
48	−0.04	−1.64	0.101	0.997	−0.03	−1.3	0.194	0.974
49	0.09	4.17	<0.000*	0.002*	0.1	4.3	<0.000*	0.001
50	0.03	1.33	0.184	0.997	−0.02	−1.07	0.286	0.974
51	0.02	0.88	0.381	0.997	0.04	1.53	0.126	0.974
52	−0.03	−1.46	0.144	0.997	0	−0.1	0.92	0.974
53	0.01	0.63	0.53	0.997	0.04	1.7	0.09	0.974
54	−0.09	−4.13	<0.000*	0.002	−0.08	−3.41	0.001*	0.041*
55	0.04	1.84	0.066	0.997	0.06	2.47	0.013*	0.791
56	0	0.18	0.856	0.997	0.01	0.65	0.516	0.974
57	0	−0.16	0.871	0.997	−0.01	−0.33	0.743	0.974
58	0.03	1.23	0.218	0.997	0.04	1.67	0.095	0.974
59	−0.33	‐	‐	‐	0.17	‐	‐	‐
60	0.05	2.25	0.025*	0.997	0.02	1.07	0.286	0.974
61	−0.03	−1.24	0.214	0.997	−0.01	−0.33	0.738	0.974
62	0.01	0.39	0.696	0.997	−0.03	−1.29	0.197	0.974
63	0.03	1.31	0.192	0.997	0.02	0.96	0.335	0.974
64	−0.04	−1.73	0.085	0.997	−0.06	−2.53	0.012*	0.694
65	−0.02	−0.73	0.465	0.997	−0.03	−1.23	0.219	0.974
66	−0.03	−1.42	0.156	0.997	−0.05	−2.26	0.024*	0.974
67	0	0.19	0.853	0.997	−0.04	−1.66	0.098	0.974
68	0.03	1.15	0.251	0.997	0.04	1.64	0.102	0.974
69	−0.01	−0.51	0.608	0.997	0	−0.18	0.855	0.974
70	0.02	0.86	0.392	0.997	0.03	1.38	0.166	0.974

*Note*: The table depicts Cohens D, T statistic, and uncorrected‐ and FDR‐corrected *p*‐values from the statistical analyses of the associations between parental education, parental income, and each component loading. Significant *p*‐values are marked with Asterix (*).

Parental education showed a positive association with IC1, which was a bi‐hemispheric component, dominated by larger global surface area (87%) and to a lesser extent lower global GWC (8%). This indicates that parental education is associated with larger cortical surface area and lower GWC in child offspring.

Parental education showed a stronger positive association with IC2 than with IC1. IC2 was a bi‐hemispheric and mostly unimodal component of higher global GWC (90%) and to a lesser extent larger surface area (7%). Relative to the findings of IC1, this indicates that parental education is somewhat linked to higher GWC globally and larger surface area in childhood.

The strongest association for parental education was a positive association with IC10. IC10 was bi‐hemispheric and multimodal, capturing a joint pattern of lower GWC (43%) and higher thickness (28%) and surface area (27%) within temporal pole and insular regions, as well as lower medial occipital thickness. This indicates that beyond the global findings already described, parental education is associated with lower GWC, thicker cortex and larger surface area in insular cortical regions in childhood.

Parental education showed a positive association with IC20, which showed a bi‐hemispheric pattern of larger occipital and smaller parietal surface area (77%) as well as an overlapping bidirectional and local thickness (11%) and GWC (8%) pattern. This indicates that parental education is linked to additional local variations in child cortical structure, particularly of larger surface area.

Parental education showed a positive association with IC49, a component with a bi‐hemispheric pattern of higher thickness (55%) within occipital, temporal and parietal regions extending into the frontal lobe, as well as a bi‐directional pattern for GWC in similar regions (40%). Parental education is therefore linked to additional local variations, particularly of higher cortical thickness and higher and lower GWC locally in childhood.

Parental education showed a negative association with IC54. It generally showed a bi‐hemispheric pattern of larger occipital and smaller frontal surface area (77%), larger medial occipital thickness (13%), and higher medial occipital‐, and lower lateral occipital GWC (10%). This suggests that parental education is linked to further local variations in child cortical structure, particularly of smaller occipital and larger frontal surface area.

In summary, our results suggest that children with more educated parents have higher GWC and larger surface area globally, as well as additional local cortical variations of larger frontal and insular surface area and to a lesser extent bidirectional GWC patterns and higher thickness.

### Linear associations between parental income and youth cortical structure

LME models revealed significant associations between parental income and 7 ICs of youth cortical structure, namely IC1, IC2, IC4, IC10, IC20, IC49 and IC54 (see Table 1 and Figure 3).

In addition to the ICs already described above, parental income showed a negative association with IC4. IC4 was dominated by larger occipito‐parietal‐ and smaller frontal surface area (89%) of the right hemisphere, and to a lesser extent higher right hemisphere frontal and occipital GWC (6%). This indicates that beyond the relations already described, parental income is associated with local, right‐hemisphere variations in child cortical structure, of smaller occipito‐parietal and larger frontal surface area, as well as lower local GWC to a lesser extent. Quadratic associations between parental income and LICA components are presented in the SI and SI Table 2. The spread of effect sizes for ICs significantly associated with SES subfactors across scanners are presented in SI Figure 8.

### Linear associations between parental SES and child p‐factor, and moderation effects of cortical structure

LME models revealed significant negative associations between the child p‐factor scores and both parental education (*d* = −0.24, *t* = −10.90, *p* = <0.001) and parental income (*d* = −0.31, *t* = −13.67, *p* = <0.001). We then tested whether the negative parental SES—p‐factor relations were moderated by individual differences in cortical structure.

**FIGURE 4:**
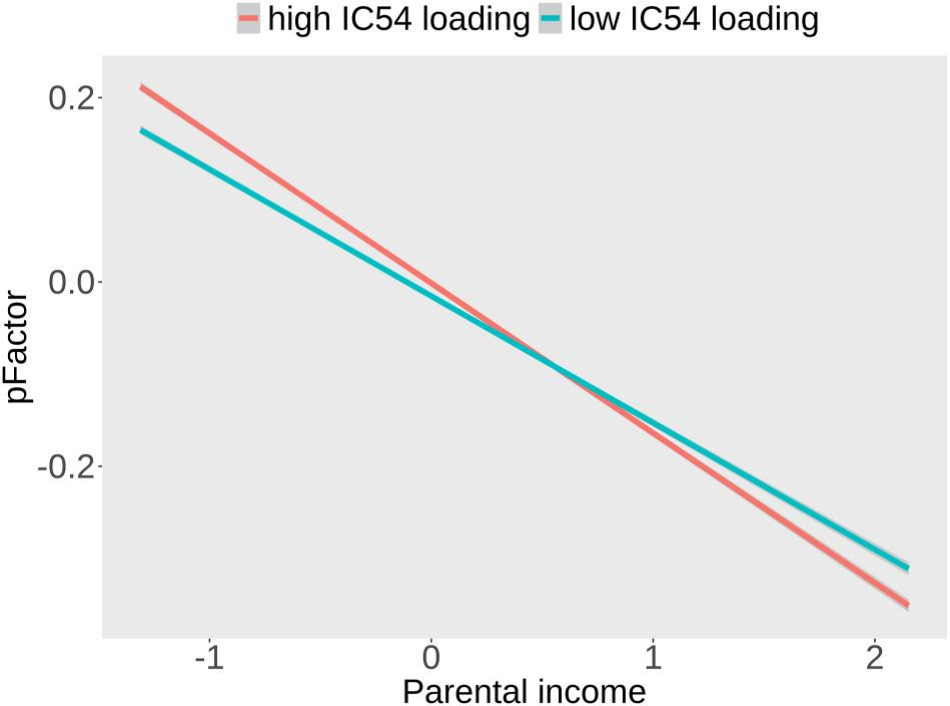
A visualization of the moderation effect of IC54 on the relationship between parental income and child p‐factor scores. For visualization, the sample is divided by median split, so that subjects who show a high loading on IC54 are marked in red, and subjects who show a low loading on IC54 are marked in green.

LME models revealed no moderation effect of cortical structure on the parental education ‐ child p‐factor relationship (SI Table 3). We found a significant negative moderation effect of IC54 (*d* = −0.07, *t* = −3.06, corrected *p* = 0.016) on the negative relationship between parental income and child p‐factor scores (Figure 4 and SI Table 3). This indicates that the relationship between parental income and psychopathology in childhood is attenuated for children with larger frontal‐, and smaller occipital surface area and lower medial occipital thickness and GWC.

### Linear associations between parental SES and unimodal global cortical metrics

To compare our findings to standard unimodal assessments, we ran LME models testing the association between parental SES and unimodal metrics, separately. These results are described in the SI and SI Table 4. In short, unimodal assessments showed close correspondence to our multimodal fusion analyses, with significant positive associations for both parental education and parental income and total surface area as well as mean GWC. We did not find any significant associations between either parental SES metric and mean cortical thickness or mean curvature.

### DISCUSSION

We sought to characterize the relationships between the socioeconomic environment and a child's cortical structure, and whether it moderated the association between parental SES and child psychopathology. Children from more educated and affluent parents had a combination of larger surface area and GWC, in addition to local variations of larger surface area and to a lesser extent bidirectional GWC and thickness patterns. The negative relationship between parental income and child psychopathology was attenuated for children showing a pattern of larger frontal and smaller occipital surface area and lower medial occipital thickness and GWC.

The multimodal decomposition of vertex‐wise cortical metrics revealed that most of the variance within the data could be explained by global surface area, and global GWC. Despite the participants' relatively young age, the prominence of global surface area could in part be due to sex‐related differences. As expected in a sample with a narrow age range, cortical thickness was less dominant, as was curvature with minimal contribution. A more detailed discussion of our FLICA decomposition is discussed within the SI.

In line with previous studies (Noble et al., 2015; Rakesh & Whittle, 2021) our results showed that parental education and income were both associated with larger cortical surface area in children. The neurobiology underlying MRI based area differences in late childhood are not fully understood. Increased pericortical myelin and axon calibers could possibly be protruding‐ and pushing the cortical surface outward, increasing its size, while at the same time decreasing cortical folding and thickness (Seldon, 2005). Indeed, although contributions were small, fused cortical thickness and GWC showed global negative patterns in accordance with a thinner and brighter cortex. Curvature, on the other hand, did not show this pattern.

Children from higher SES families also showed globally higher GWC, emerging as the strongest finding for parental income and, apart from a multimodal insular pattern, also for parental education. While it can be challenging to interpret separate components with opposing patterns, our unimodal analyses concordantly showed a positive association between both parental SES metrics and global GWC. There has to our knowledge been no previous tests of the relations between parental SES and child GWC. However, our findings correspond well with a recent paper that assessed parental SES and its associations to the related, but directionally inverse, intensity metric T1w/T2w ratio (Norbom et al., 2022). Here, widespread negative relations were found between general‐ and subfactor parental SES and T1w/T2w ratio. Effect sizes were also larger than for standard morphometry, including cortical surface area and cortical thickness. GWC thus appears to be a highly sensitive imaging marker not only for youth cortical development generally (Norbom et al., 2019, 2020), but also specifically for SES related variance.

From a neurobiological standpoint, although consistently reported and thus in line with our hypothesis, the direction of GWC results is counterintuitive. GWC decreases across childhood and adolescence, pointing to gray and white matter intensities becoming more similar, possibly due to higher levels of intracortical myelin. Our results may thus indicate that children from lower SES families have a more developed cortex. Possible reasons for the direction of findings are discussed in detail elsewhere (Norbom et al., 2022), but include a disadvantageous effect of excess myelin, cortical surface misclassification, and other tissue properties accounting for GWC variations. On the other hand, our results fit with the theory that exposure to poverty and early adversity can foster accelerated maturation (Belsky, 2019; Colich et al., 2020). This notion has also been supported by MRI based studies (Colich et al., 2020). It would be beneficial to employ quantitative relaxometry to further probe the underlying neurobiology linking SES to GWC.

Multimodal fusion revealed that parental education and income were associated with complex multimodal cortical patterns, including larger frontal and insular surface area, coupled with lower insular GWC and thickness. Although previous research is discrepant, the most consistent morphometric pattern is indeed a positive association between parental SES and frontal surface area (Rakesh & Whittle, 2021). Although based on large tissue volumes, overlapping multimodal patterns should improve our inferences of the underlying neurobiology. For instance, children from higher‐income parents had right hemisphere increases in frontal surface area weakly coupled with thinner cortex and lower GWC. Similarly, our insular component coupled larger surface area with lower GWC. In youth MRI studies, these are well documented maturational patterns (Norbom et al., 2021) that in sum are understood to reflect a combination of pericortical and cortical myelination, increased axon caliber, remodeling of dendritic arbor and reductions in glial cells (Huttenlocher & Dabholkar, 1997; Petanjek et al., 2008, 2011; Peter R., 1979; Seldon, 2005; Vidal‐Pineiro et al., 2020). While structure to function relations are not analogous, future fMRI studies could assess whether the socioeconomic environment also affects frontal cortex and insular function.

As expected, parental SES was not closely linked to cortical thickness in youth, only showing a few highly local variations within multimodal components. Findings were corroborated by unimodal mean thickness analyses showing no significant associations to either parental SES metric. This is in line with previous research pointing to mixed‐ and mostly null findings regarding parental SES and cortical thickness (Rakesh & Whittle, 2021). Yet, our observations underscore the advantage of multimodal fusion in discerning subtle patterns that are not captured in unimodal thickness assessments. Overall, our findings could indicate that cortical thinning across youth might be affected more by genetic factors, while surface area (Strike et al., 2019) and GWC could be more sensitive to environmental impact. However, a recent ABCD study found a relation between family income and ROI based cortical thickness (Tomasi & Volkow, 2021), as did several papers testing the related concept of neighborhood disadvantage (Rakesh & Whittle, 2021; Taylor et al., 2020; Vargas et al., 2020). A similar study by Hackman et al. (2021) did not corroborate these findings.

Multi‐ and unimodal assessments did not reveal an association between child socioeconomic environment and cortical curvature. Developmental folding differences across youth have been less explored, and to our knowledge no studies have previously assessed the SES ‐ youth curvature relation. Nevertheless, a multivariate brain structure–behavior mapping revealed several modes of covariation, including one capturing general economic deprivation, and the related metric sulcal depth (Alnæs et al., 2020).

In line with our hypothesis and current literature (Letourneau et al., 2013; Reiss, 2013) children from less educated and lower income families were reported to have higher levels of general psychopathology. We tested whether cortical macro‐, and microstructure moderate this relationship. We found a small moderation indicating that in low‐income families child psychopathology were attenuated in children with a multimodal pattern of larger frontal‐ and smaller occipital surface area, and lower medial occipital thickness and GWC. Within higher income families where general psychopathology levels are low, we found the reverse pattern. Still, this finding should be interpreted with caution awaiting replication. Future studies should also employ longitudinal designs to test whether cortical structure mediates the relation between parental SES and future psychopathology in youth (Farah, 2017; Maxwell & Cole, 2007).

There are several limitations to our study. First, regarding our SES estimations, we did not include subjective or cultural aspects of the construct (W. M. Liu et al., 2004; Yosso, 2005) nor did we investigate the unique contributions of our SES metrics. Also, income and education are distal markers for proximal causal pathways including stress, cognitive stimulation, obstetric complications, prenatal care, toxins and nutrition (Evans & Kim, 2013; Farah, 2017; Thomas & Coecke, 2023). Second, our study design can only capture correlational relationships and cannot infer causation. Similarly, longitudinal MRI data is needed to test the maturation of cortical structure and its relation to SES over time. Third, several brain related findings including the moderation effect, were of small magnitude. This is not surprising as publication bias and historically small samples may have promoted inflated neurodevelopmental effects (Button et al., 2013; Ioannidis, 2008) that should be attenuated when assessing ABCD (Dick et al., 2021). Also, for many outcomes in nature causal relations are in reality small (Dick et al., 2021), including clinical effects within psychology and psychiatry (Meyer et al., 2001), and in multimodal imaging and health outcomes (Miller et al., 2016).

It is presently unclear *how* childhood socioeconomic diversity becomes neurobiologically embedded and influence current and future risk for mental health problems. In the present study we report that children with more educated or affluent parents have a combination of larger global and regional surface area, a larger difference between gray and white matter intensities, and that in low‐income families, having what appears to be a more developed cortex across metrics is beneficial to mental health.

## AUTHOR CONTRIBUTION

**Linn B. Norbom**: Conceptualization, Formal analysis, Investigation, Methodology, Visualization, Writing – original draft, Writing – review & editing. **Jaroslav Rokicki**: Methodology, Supervision, Writing – review & editing. **Espen M. Eilertsen**: Formal analysis, Methodology, Writing – review & editing. **Thea Wiker**: Formal analysis, Methodology, Writing – review & editing. **Jamie Hanson**: Conceptualization, Methodology, Writing – review & editing. **Andreas Dahl**: Methodology, Writing – review & editing. **Dag Alnæs**: Methodology, Writing – review & editing. **Sara Fernández‐Cabello**: Methodology, Writing – review & editing. **Dani Beck**: Writing – review & editing. **Christian K. Tamnes**: Conceptualization, Funding acquisition, Investigation, Methodology, Project administration, Resources, Supervision, Visualization, Writing – original draft, Writing – review & editing.

## CONFLICT OF INTEREST STATEMENT

The authors have declared they have no competing or potential conflicts of interest.

## ETHICAL CONSIDERATIONS

As specified within supplemental information parental informed consent as well as child assent was obtained for all individuals. The Institutional Review Board at the University of California, San Diego, approved all aspects of ABCD Study® (Auchter et al., 2018). The current study was conducted in line with the Declaration of Helsinki and was approved by the Regional Committee for Medical and Health Research Ethics (REK 2019/943).

## Supporting information

Supporting Information S1

## Data Availability

The data can be acquired from the ABCD Study® (https://data‐archive.nimh.nih.gov/abcd). The statistical code used in the present paper can be found online (https://osf.io/etsjx/).
